# A prospective cohort study of presenteeism and poverty among Japanese workers during the COVID‐19 pandemic

**DOI:** 10.1002/1348-9585.12342

**Published:** 2022-07-05

**Authors:** Yoshihisa Fujino, Makoto Okawara, Yu Igarashi, Mami Kuwamura, Ayako Hino, Keiji Muramatsu, Tomohisa Nagata, Akira Ogami, Tomohiro Ishimaru, for the CORoNaWork project

**Affiliations:** ^1^ Department of Environmental Epidemiology Institute of Industrial Ecological Sciences, University of Occupational and Environmental Health, Japan Kitakyushu Japan; ^2^ Disaster Occupational Health Center Institute of Industrial Ecological Sciences, University of Occupational and Environmental Health, Japan Kitakyushu Japan; ^3^ Department of Environmental Health, School of Medicine University of Occupational and Environmental Health, Japan Kitakyushu Japan; ^4^ Department of Mental Health Institute of Industrial Ecological Sciences, University of Occupational and Environmental Health, Japan Kitakyushu Japan; ^5^ Department of Preventive Medicine and Community Health, School of Medicine University of Occupational and Environmental Health, Japan Kitakyushu Japan; ^6^ Department of Occupational Health Practice and Management Institute of Industrial Ecological Sciences, University of Occupational and Environmental Health, Japan Kitakyushu Japan; ^7^ Department of Work Systems and Health Institute of Industrial Ecological Sciences, University of Occupational and Environmental Health, Japan Kitakyushu Japan

**Keywords:** Japan, poverty, presenteeism, work performance, working poor

## Abstract

**Objectives:**

This study examined the association of presenteeism with experiences of poverty among Japanese workers during the COVID‐19 pandemic.

**Methods:**

A prospective cohort study of Japanese workers was conducted using an Internet monitoring survey. The baseline survey was conducted in December 2020, and a follow‐up survey in December 2021. Of the 27 036 workers who participated, 18 560 (68.7%) completed the follow‐up survey. The 11 081 who reported that they were not in financial difficulty in the baseline survey were included in the analysis. The degree of work functioning impairment was assessed at baseline using the Work Functioning Impairment Scale (WFun). Households' experience of not being able to pay for food and clothing was identified in the follow‐up survey. The odds ratios (ORs) of presenteeism determined by WFun associated with poverty were estimated using a multilevel logistic model. The multivariate model included age, sex, marital status, job type, income, education, smoking, alcohol consumption, number of employees in the workplace, and the incidence rate of COVID‐19 by prefecture at baseline.

**Results:**

In the multivariate model, the odds ratio of experiencing food insecurity increased with high WFun score: compared with WFun scores of 13 or less, the OR was 1.87 (95% CI: 1.43–2.43, *P* < .001) for WFun scores of 14 or more and 3.26 (95% CI: 2.58–4.12, *P* < .001) for WFun scores of 21 or more.

**Conclusions:**

In addition to labor productivity, the adverse effects of presenteeism on social security‐related concerns such as poverty require further attention.

## 1 INTRODUCTION

Presenteeism is a condition in which a worker continues to work while feeling unwell.^1^ Presenteeism is increasingly being recognized as a business concern because it can reduce labor productivity and performance.^2^ Simultaneously, presenteeism is a medium‐ to long‐term health risk for workers because it is associated with delayed treatment and worsening of symptoms, and is considered a barrier to quality of life and continued employment.^3^, ^4^, ^5^ Workers with presenteeism are reportedly more likely to take long‐term leaves of absence and to retire early.^4^, ^5^, ^6^, ^7^, ^8^ Such employment disadvantages are important public health issues that lead to economic deprivation not only for individual workers, but also their families; this in turn can develop into an important social security concern.

For many people, the primary purpose of work is to earn an income to sustain their daily lives and provide for their households. People with presenteeism experience work disability, or the inability to perform certain tasks such as work necessitating physical strength, shift work, and business trips.^9^, ^10^, ^11^ Workers who experience work disabilities gradually reduce their work hours and change their job description, workplace, and even employment.^9^, ^12^ These changes often lead to unstable employment and downward earnings. In addition to employment disadvantage, workers with presenteeism can be at risk of household hardship, or poverty.

Poverty is an emerging and long underestimated social issue in Japan, despite the country being the second richest in the world from 1968 to 2009 and currently the third richest according to the Gross Domestic Product^13^ Japanese companies have long adopted a lifetime employment system and salary system based on seniority, which have served as the social foundation upon which the middle class is supported.^14^ The rise in non‐regular employment, especially among young people, in recent years, however has led poverty to become increasingly recognized as a social problem.^15^ An estimated 36.7% of all workers are thought to have informal employment,^16^ and Japan was ranked 22nd in 2020 for average wage among Organization for Economic Co‐operation and Development countries.^17^ The relative poverty rate in Japan was 15.3% in 2000.^18^

The global COVID‐19 pandemic has affected both the employment and presenteeism‐related experience of workers, and has resulted in precarious employment conditions in Japan.^18^ The pandemic has also caused interruptions to treatment for workers with chronic illnesses and increased anxiety due to infection.^19^ Lockdowns and social distance mandates have additionally brought about challenges such as loneliness and stress.^20^, ^21^ These events have further increased presenteeism among workers, especially those in precarious socioeconomic situations, who are more likely to experience presenteeism.^22^, ^23^, ^24^ Workers with presenteeism may be at high risk of poverty, as they are more likely to experience a decline in income due to reduced working hours, changes to less demanding tasks, and transitions to more precarious forms of employment. However, while there have been reports of an association between presenteeism and long‐term absence from work and early retirement,^21^ to our knowledge, there are no reports of an association between presenteeism and poverty, either relative or absolute.

We hypothesized that workers with presenteeism are at high risk of poverty. To test this theory, we conducted a cohort study to examine the relationship between symptoms affecting an individual's ability to work, the degree of work dysfunction, and the level of household financial difficulty.

## 2 MATERIALS AND METHODS

A prospective cohort study was conducted based on data obtained through surveys administered over the Internet; a baseline survey was conducted in December 2020 and a follow‐up survey 1 year later in December 2021. All participants provided informed consent and the study was approved by the Ethics Committee of University of Occupational and Environmental Health, Japan (Ref. Nos. R2‐079 and R3‐006).

The study was conducted according to a previously detailed protocol.^25^ The target population consisted of participants who had preregistered with an online survey research company. Requirements for participation in the survey were workers between the ages of 20 and 65 at baseline. Stratified sampling was conducted among subjects who met the inclusion criteria, grouped by sex, job category, and geographic region. Further, Japan's 47 prefectures were classified into five regions based on the cumulative incidence of COVID‐19. Specifically, 20 blocks with an approximately equal number of participants were created by combining participants with different sex, occupation (office and non‐office workers), and region (five categories). We planned to enroll 1500 participants in each block for a total of 30 000 participants.

The survey was commissioned to Cross Marketing Inc. Approximately 600 000 of the company's 4.7 million preregistered users were sent an e‐mail requesting their participation in the survey. Of these, 55 045 participated in the primary screening and 33 302 met the final eligibility criteria. Of these 33 302, those who were determined to be unreliable respondents were excluded, leaving 27 036 for analysis. Criteria used to determine unreliable responses included extremely short response times (less than 6 minutes), extremely low weight (less than 30 kg), extremely low height (less than 140 cm), inconsistent responses to similar questions throughout the survey (e.g., questions about marital status and region of residence), and questions used to identify fraudulent responses. Since the survey system required all questions to be answered, there were no missing values. At the 1‐year follow‐up, 18 560 (68.7%) of the original respondents participated.

### 2.1 Data retrieval

At baseline, we asked the following question about participants' economic situation: “How do you feel about your current financial situation?” Participants chose from five options: “very distressed,” “somewhat distressed,” “neither distressed nor happy,” “somewhat happy,” and “very happy.” Those who responded, “very distressed” (222, 1%) or “somewhat distressed” (5257, 28%) were excluded, and those who responded “neither distressed nor happy,” (8796, 47%) “somewhat happy,” (1962, 11%) or “very happy” (323, 2%) were included in the analysis in order to restrict the subject as population at risk for poverty. A total of 11 081 respondents were included in this study.

### 2.2 Evaluation of health condition and work functioning impairment

At baseline, we identified the health conditions that were affecting participants' ability to work using two methods. In the first method, we asked them about their symptoms known to be frequently associated with presenteeism in previous studies: “Which of the following is closest to the health problem that is most affecting your work?” They answered by selecting one of the following options: “No particular problems”; “Pain‐related problems”; “Physical movement and mobility”; “Fatigue, loss of strength, appetite, fever, dizziness, and moodiness”; “Toileting and defecation”; “Mental health problems”; “Skin, hair, and cosmetic concerns”; “Sleep”; “Eye‐related matters”; “Nasal matters”; “Hearing”; and “Other”.

In the second method, we measured the degree of work function impairment using The Work Functioning Impairment Scale (WFun).^26^ WFun is a self‐reported outcome measure developed based on the Rash model that has been validated based on consensus‐based standards for the selection of health measurement instruments (COSMIN). It consists of the following seven statements: “I haven't been able to behave socially”; “I haven't been able to maintain the quality of my work”; “I have had trouble thinking clearly”; “I have taken more rests during my work”; “I have felt that my work isn't going well”; “I haven't been able to make rational decisions”; and “I haven't been proactive about my work.” The participant responds to each statement on a five‐point scale: 1 = “not at all,” 2 = “one or more days a month,” 3 = “about one day a week,” 4 = “two or more days a week” and 5 = “almost every day.” The WFun score thus ranges from 7 to 35 points, with a higher score indicating a greater degree of work functioning impairment. Based on previous studies, a WFun score of 14 to 20 was used to indicate moderately impaired work function and a score of 21 or higher to indicate severely impaired work function.^27^

### 2.3 Assessment of financial distress

This study used the level of financial difficulty within each household as an indicator of poverty, as is commonly used in poverty surveys in Japan.^28^ Financial difficulty is measured based on a household's experience of being unable to purchase the required food and clothing. Household financial distress was investigated at the follow‐up survey using two questions. The first asked about the household's experience with not being able to afford food, and the second asked about the household's experience with not being able to afford clothing. The questions were as follows: “In the past year, has your household been unable to buy the food your family needs due to lack of money? Note that this does not include luxury items” and “In the past year, has your household been unable to buy the clothing your family needs due to lack of money? Note that this does not include expensive clothing or precious metals/jewelry.” Subjects responded by choosing from four options: “often,” “sometimes,” “rarely,” and “never.” Those who chose either “often” or “sometimes” were defined as experiencing food or clothing insecurity.

### 2.4 Other covariates

Participants provided the following information in the baseline survey: age, sex, prefecture of residence, marital status (unmarried, bereaved/divorced), job type (mainly desk work, mainly interpersonal communication, and mainly labor), number of employees in the workplace (1–4, 5–49, 50–499, >500), educational background (Junior high school, high school, vocational school/college/university/graduate school), equivalent household income (household income divided by the square root of household size), smoking status (current smoker, non‐current smoker), and alcohol consumption (6–7 days a week, 4–5 days a week, 2–3 days a week, less than 1 day a week, hardly ever).

### 2.5 Statistical analyses

In the analyses, health condition and work functioning impairment were treated as the exposure variables, and financial distress was treated as the outcome variable. The odds ratios (ORs) of experiencing food and clothing insecurity associated with each work function‐related symptom were estimated using a multilevel logistic model, which was nested in the prefecture of residence to account for regional differences. We also estimated ORs of experiencing food and clothing insecurity associated with work functioning impairment according to the WFun scale.

Further, age‐sex‐adjusted and multivariate‐adjusted ORs were estimated. The multivariate model included age, sex, marital status, job type, equivalent household income, education, smoking, alcohol consumption, number of employees in the workplace, and the incidence rate of COVID‐19 by prefecture at baseline. A *P* value less than .05 was considered statistically significant. All analyses were conducted using Stata (Stata Statistical Software: Release 17; StataCorp LLC).

## 3 RESULTS

Table 1 shows participants' baseline characteristics by WFun score. Compared to those with a WFun score of 13 or less, participants with a WFun score of 21 or more were older and less likely to be male. While the percentage of subjects in the lowest equivalent household income category (below 3 million yen) did not differ between the WFun groups, 32.3% of those with a WFun score of 13 points or less had equivalent household income above 8 million yen compared to 27.5% of participants with a WFun of 21 points or more. There were no significant differences in smoking or drinking habits between the WFun groups.

**TABLE 1:** Baseline characteristics according to participants' WFun score

	Work functioning impairment scale		
	7–13 *n* = 7389	14–20 *n* = 1976	21–35 *n* = 1716
Age, median (interquartile range)	50.0 (43.0, 57.0)	49.0 (40.0, 56.0)	47.0 (37.0, 54.0)
Sex, male	4239 (57.4%)	1009 (51.1%)	876 (51.0%)
Marital status			
Married	4472 (60.5%)	1146 (58.0%)	912 (53.1%)
Education			
Junior high school	5622 (76.1%)	1545 (78.2%)	1370 (79.8%)
High school	1699 (23.0%)	415 (21.0%)	336 (19.6%)
Vocational school/ College/ university/ Graduate school	68 (0.9%)	16 (0.8%)	10 (0.6%)
Job type, mainly desk work Income (million JPY)	4079 (55.2%)	1098 (55.6%)	966 (56.3%)
<300	1176 (15.9%)	285 (14.4%)	290 (16.9%)
300–499	2357 (31.9%)	673 (34.1%)	602 (35.1%)
500–799	1480 (20.0%)	418 (21.2%)	352 (20.5%)
>800	2376 (32.2%)	600 (30.4%)	472 (27.5%)
Number of employees in the workplace			
1–4	1640 (22.2%)	346 (17.5%)	239 (13.9%)
5–49	1843 (24.9%)	490 (24.8%)	457 (26.6%)
50–499	1853 (25.1%)	551 (27.9%)	497 (29.0%)
>500	2053 (27.8%)	589 (29.8%)	523 (30.5%)
Current smoker	1871 (25.3%)	510 (25.8%)	400 (23.3%)
Alcohol consumption			
6–7 days per week	1746 (23.6%)	431 (21.8%)	369 (21.5%)
4–5 days per week	623 (8.4%)	173 (8.8%)	124 (7.2%)
2–3 days per week	915 (12.4%)	260 (13.2%)	228 (13.3%)
Less than 1 day per week	1196 (16.2%)	358 (18.1%)	287 (16.7%)
Hardly ever	2909 (39.4%)	754 (38.2%)	708 (41.3%)

Table 2 shows the frequency of difficulties experienced by households by WFun score and symptom. Of the 11 081 subjects, 3.6% indicated that they had experienced difficulty buying the food they needed, and 3.7% indicated difficulty buying the clothing during the 1‐year follow‐up period. Of all, 327 (3.0%) reported they had experienced difficulties buying both food and clothing. A high WFun score was related to a greater proportion of workers experiencing difficulties buying food: 2.4% of those with a WFun score of 13 or less experienced hardship related to purchasing food compared to 8.4% of participants with a WFun score of 21 or more. By symptom, the greatest percentage of people reporting food insecurity had mobility‐related issues (8.7%). This was followed by those with mental health problems (5.9%) and those with chronic fatigue (5.7%). Similar findings were obtained for those experiencing difficulties buying clothing.

**TABLE 2:** Frequency of poverty‐related experiences by WFun score and symptom

	*n*	Food deprivation *n* (%)	Clothing deprivation *n* (%)
Total	11 081	404 (3.6%)	411 (3.7%)
Food deprivation	404	‐	327 (79.6%)
Clothing deprivation	411	327 (80.9%)	‐
Work functioning impairment scale 7–13	7389	176 (2.4%)	179 (2.4%)
14–20 (moderately impaired)	1976	89 (4.5%)	88 (4.5%)
21–35 (severely impaired)	1716	139 (8.1%)	144 (8.4%)
Symptoms affecting participants' ability to work.			
No problem	7138	211 (3.0%)	219 (3.1%)
Pain‐related problems	604	24 (4.0%)	25 (4.1%)
Physical movement and mobility	265	23 (8.7%)	23 (8.7%)
Fatigue, loss of strength, appetite, fever	385	22 (5.7%)	23 (6.0%)
Toileting and defecation	161	7 (4.3%)	8 (5.0%)
Mental health problems	648	38 (5.9%)	38 (5.9%)
Skin, hair, and cosmetic concerns	188	10 (5.3%)	9 (4.8%)
Sleep	587	30 (5.1%)	28 (4.8%)
Eye‐related matters	506	20 (4.0%)	18 (3.6%)
Nasal matters	68	3 (4.4%)	2 (2.9%)
Hearing	67	2 (3.0%)	2 (3.0%)
Other	464	14 (3.0%)	16 (3.4%)

Table 3 shows the odds ratios of experiencing food insecurity associated with work functioning impairment determined by WFun and symptoms affecting participants' ability to work. In the age‐sex‐adjusted model, the odds ratio of experiencing food insecurity was higher with increasing WFun score: compared with WFun scores of 13 or less, the OR was 1.86 for WFun scores of 14 or more and 3.22 for WFun scores of 21 or more. In terms of symptoms identified as affecting participants' ability to work, the OR of experiencing food insecurity was highest at 3.13 for mobility‐related problems, followed by 1.87 for chronic fatigue and 1.83 for mental health problems. Model 2, which adjusted for confounding factors such as socioeconomic factors, produced the same results regardless of sex and age. However, in Model 3, where WFun score and symptoms affecting participants' ability to work were included simultaneously, WFun score and mobility‐related symptoms were still associated with experiencing food insecurity, but the other symptoms were not. Further, the OR value was reduced compared to those in Model 1 and Model 2 (OR = 2.34, 95% CI 1.46–3.75, *P* < .001).

**TABLE 3:** Odds ratios of experiencing food insecurity associated with WFun score and symptoms affecting participants' ability to work

	Age‐sex adjusted				Model 1^b^				Model 2^c^			
	OR	95% CI		*P*	OR	95% CI		*P*	OR	95% CI		*P*
Work functioning impairment scale												
7–13	Reference				Reference				Reference			
14–20 (moderately impaired)	1.86	1.43	2.42	<.001	1.87	1.43	2.43	<.001	1.71	1.30	2.25	<.001
21–35 (severely impaired)	3.22	2.55	4.06	<.001 <.001^a^	3.26	2.58	4.12	<.001 <.001^a^	3.01	2.34	3.87	<.001 <.001^a^
Symptoms affecting participants' ability to work												
No problem	Reference				Reference				Reference			
Pain‐related problems	1.45	0.94	2.24	.092	1.42	0.92	2.20	.112	1.12	0.72	1.74	.623
Physical movement and mobility	3.13	1.99	4.94	<.001	3.23	2.04	5.11	<.001	2.34	1.46	3.75	<.001
Fatigue, loss of strength, appetite, fever	1.87	1.19	2.96	.007	1.87	1.18	2.96	.008	1.26	0.79	2.02	.336
Toileting and defecation	1.53	0.70	3.33	.282	1.40	0.64	3.06	.402	1.11	0.51	2.44	.791
Mental health problems	1.83	1.27	2.62	.001	1.87	1.30	2.70	.001	1.15	0.78	1.68	.482
Skin, hair, and cosmetic concerns	1.59	0.82	3.07	.172	1.60	0.82	3.11	.166	1.30	0.66	2.55	.441
Sleep	1.65	1.11	2.46	.013	1.62	1.08	2.41	.019	1.17	0.77	1.76	.460
Eye‐related matters	1.62	1.01	2.60	.045	1.73	1.07	2.78	.024	1.38	0.85	2.23	.192
Nasal matters	1.65	0.51	5.33	.403	1.62	0.50	5.25	.424	1.31	0.40	4.29	.654
Hearing	1.05	0.25	4.34	.950	1.05	0.25	4.38	.946	0.82	0.19	3.46	.783
Other	1.00	0.58	1.74	.992	1.01	0.58	1.76	.970	0.83	0.47	1.45	.506

^a^ *P* for trend.

^b^ The model included age, sex, marital status, job type, income, education, smoking, alcohol consumption, number of employees in the workplace, and the incidence rate of COVID‐19 by prefecture at baseline.

^c^ The model included WFun and symptoms simultaneously, adjusting for age, sex, marital status, job type, income, education, smoking, alcohol consumption, number of employees in the workplace, and the incidence rate of COVID‐19 by prefecture at baseline.

Table 4 shows the OR of experiencing difficulties purchasing clothing associated with WFun score and symptoms affecting participants' ability to work. The results were almost identical to those obtained for food insecurity. The sex‐ and age‐adjusted models and the model that adjusted for socioeconomic factors showed significant associations between difficulties purchasing clothing and WFun score, mobility‐related symptoms, chronic fatigue, and mental health problems. However, in the model that included both the WFun score and symptoms affecting participants' ability to work, WFun score and mobility‐related symptoms were still associated with experiencing difficulty buying clothing, but the other symptoms were not: the OR for those with a WFun score of 14 or more and 21 or more points was 1.71 and 3.13, respectively, compared to the group with a WFun score of 13 points or less.

**TABLE 4:** Odds ratios of experiencing difficulties purchasing clothing associated with WFun score and symptoms affecting participants' ability to work

	Age‐sex adjusted				Model 1^b^				Model 2^c^			
	OR	95% CI		*P*	OR	95% CI		*P*	OR	95% CI		*P*
Work functioning impairment scale												
7–13	Reference				Reference				Reference			
14–20 (moderately impaired)	1.81	1.39	2.35	<.001	1.82	1.40	2.37	<.001	1.71	1.30	2.25	<.001
21–35 (severely impaired)	3.29	2.61	4.13	<.001 <.001^a^	3.32	2.63	4.18	<.001 <.001^a^	3.13	2.44	4.01	<0.001 <.001^a^
Symptoms affecting participants' ability to work												
No problem	Reference				Reference				Reference			
Pain‐related problems	1.46	0.95	2.23	.081	1.43	0.93	2.19	.105	1.12	0.72	1.73	.623
Physical movement and mobility	3.02	1.92	4.76	<.001	3.12	1.97	4.92	<.001	2.23	1.40	3.57	.001
Fatigue, loss of strength, appetite, fever	1.87	1.20	2.93	.006	1.89	1.20	2.97	.006	1.26	0.79	2.00	.336
Toileting and defecation	1.72	0.83	3.57	.144	1.56	0.75	3.24	.239	1.24	0.59	2.60	.566
Mental health problems	1.74	1.22	2.50	.002	1.79	1.25	2.57	.002	1.08	0.73	1.58	.707
Skin, hair, and cosmetic concerns	1.31	0.66	2.61	.446	1.33	0.66	2.66	.422	1.07	0.53	2.17	.845
Sleep	1.46	0.97	2.19	.067	1.45	0.96	2.18	.076	1.03	0.68	1.57	.882
Eye‐related matters	1.41	0.86	2.30	.177	1.49	0.91	2.45	.115	1.17	0.71	1.93	.548
Nasal matters	1.04	0.25	4.31	.952	1.02	0.25	4.21	.982	0.80	0.19	3.36	.765
Hearing	1.07	0.26	4.40	.931	1.07	0.26	4.43	.929	0.83	0.20	3.48	.795
Other	1.11	0.66	1.86	.705	1.14	0.67	1.92	.633	0.92	0.54	1.56	.759

^a^ *P* for trend.

^b^ The model included age, sex, marital status, job type, income, education, smoking, alcohol consumption, number of employees in the workplace, and the incidence rate of COVID‐19 by prefecture at baseline.

^c^ The model included WFun and symptoms simultaneously, adjusting for age, sex, marital status, job type, income, education, smoking, alcohol consumption, number of employees in the workplace, and the incidence rate of COVID‐19 by prefecture at baseline.

## 4 DISCUSSION

This study revealed that Japanese workers with presenteeism experienced poverty during the COVID‐19 pandemic. The risk of poverty was particularly high among those with mobility difficulties, fatigue, mental health problems, insomnia, and eye‐related symptoms. Additionally, higher work functioning impairment tend to lead to higher risk of experiencing poverty.

There are many possible mechanisms through which workers with presenteeism experience poverty. Workers with health problems experience work disability that prevents them from performing certain tasks, including work necessitating physical strength and shift work, among others.^4^, ^5^, ^6^, ^7^, ^8^, ^9^, ^12^ Workers who experience such work disability often reduce their work hours and change job descriptions and workplaces. These lead to employment disadvantages, long‐term leaves of absence, and unemployment, which in turn lead to reduced earnings.^15^ In addition, workers with presenteeism conditions have to reduce their work hours due to hospital visits, with medical expenses forming a burden on the household budget. COVID‐19 has had a significant adverse impact on the economy and employment.^18^ It is possible that workers with presenteeism experience greater employment‐related disadvantages during such disasters. Indeed, this study confirmed the presence of an association between health status and poverty, even after controlling for socioeconomic‐related variables, such as equivalent household income and company size, which are risk factors for poverty. These findings imply that workers with presenteeism conditions could be at greater risk of poverty, leading them to experience double the burden of healthy workers.

A notable finding in this study was that general work functioning impairment was important for the risk of poverty regardless of the type of symptom affecting participants' ability to work. This was confirmed by our finding that work functioning impairment showed a significant association with food and clothing insecurity after adjusting for both work functioning impairment and symptoms simultaneously, while most symptoms, with the exception of mobility‐related problems, have disappeared to be associated with poverty experience. This result is reasonable given that a worker's ability to perform his or her job is not only determined by the type and severity of their illness or condition, but is also affected by the nature and demands of the job, along with the support of co‐workers and company systems.^29^, ^30^ Mismatches between these variables and worker's abilities and needs can reduce their capacity to perform their work. In this study, we were unable to ascertain the level of company support, workers' right to sick leave, whether telecommuting was available, or workers' level of employment security. However, workers with poor health present a degree of work functioning impairment that reflects these factors. Therefore, our findings indicate that the degree of work functioning impairment was more clearly related to experiencing poverty than work‐related symptoms.

Interest in presenteeism from a labor productivity perspective is growing in Japan, having been motivated by the so‐called “Health and Productivity Management Strategy” established by the Ministry of Economy, Trade and Industry.^31^ However, little emphasis has been placed on sickness presenteeism as a health risk behavior that can lead to further health problems and employment instability.^15^ Vulnerable socioeconomic conditions also contribute to sickness presenteeism, which can increase the risk of long‐term absences from work and unemployment. In addition, this study showed that workers with presenteeism conditions were at increased risk of experiencing poverty. While relative poverty refers to living below the average standard of living in a particular society or region, material deprivation, which refers to the inability to buy food or pay for clothing, is a level of poverty that threatens survival. The fact that workers in a wealthy country like Japan who are not yet on welfare or other forms of public assistance experience poverty related to food purchases is easily overlooked and deserves careful attention. In particular, we argue that there is a need to promote discussion on not only labor productivity, but also social security and health inequality, among workers with health problems.

There are several limitations to this study. First, this study used self‐reports of poverty‐related experiences. However, unlike asset status and income, poverty‐related experience can only be ascertained by subjective report; to our knowledge, no other method has been established for ascertaining poverty‐related experience. Further, the questions used in this study are the same as those commonly used in government statistics in Japan.^28^ Second, while this study examined income, we did not distinguish whether it was obtained from employment or capital gains. However, for a person with capital gains who is unable to work due to health reasons, the association between work functioning impairment and poverty should be low. Thus, the association observed in this study may be stronger than presented. Third, this study was conducted during a period of increasing infection rates in Japan during the COVID‐19 pandemic. Because this period overlapped with a time during which economic and employment conditions were particularly precarious,^18^ the association between health and poverty‐related experience observed in this study may be different and more emphatic than that before the COVID‐19 pandemic. Fourth, as with all prospective designs, this study only examined health status and work functioning impairment at baseline. As an improvement in health during the follow‐up period is expected lead to an attenuation of the association between health and poverty, the results presented in this study may be an underestimate.

The present study showed that workers with health problems that interfere with their work were more likely to experience poverty during the COVID‐19 pandemic than those without. The findings also showed that the association was not symptom specific, but dependent on workers' degree of work functioning impairment. Thus, in addition to labor productivity, the adverse effects of presenteeism on social security‐related concerns such as poverty require further attention. Additional discussions on support are needed among workers themselves, companies, and governments.

## AUTHOR CONTRIBUTIONS

Yoshihisa Fujino: Conceptualization, Methodology, Software, Formal analysis, Investigation, Data curation, Writing ‐ Original draft preparation, Project administration, Funding acquisition. Makoto Okawara: Investigation, Data curation, Writing ‐ Review & Editing. Yu Igarashi: Investigation, Data curation, Writing ‐ Review & Editing. Mami Kuwamura: Investigation, Data curation, Writing ‐ Review & Editing. Ayako Hino: Investigation, Data curation, Writing ‐ Review & Editing. Keiji Muramatsu: Investigation, Data curation, Writing ‐ Review & Editing. Tomohisa Nagata: Investigation, Data curation, Writing ‐ Review & Editing, Funding acquisition. Akira Ogami: Investigation, Data curation, Writing ‐ Review & Editing, Funding acquisition. Tomohiro Ishimaru: Investigation, Data curation, Writing ‐ Review & Editing.

## FUNDING INFORMATION

This study was supported and partly funded by a research grant from the University of Occupational and Environmental Health, Japan (no grant number); Japanese Ministry of Health, Labour and Welfare (H30‐josei‐ippan‐002, H30‐roudou‐ippan‐007, 19JA1004, 20JA1006, 210301‐1, and 20HB1004); Anshin Zaidan (no grant number); the Collabo‐Health Study Group (no grant number) and Hitachi Systems, Ltd. (no grant number), and scholarship donations from Chugai Pharmaceutical Co., Ltd. (no grant number). The funders were not involved in the study design, collection, analysis, interpretation of data, writing of this article or the decision to submit it for publication.

## CONFLICT OF INTEREST

Dr. Fujino has the copyright to WFun with royalties paid from Sompo Health Support Inc., outside from this work. The other authors declare no conflict of interest associated with this manuscript.

## Data Availability

The data that support the findings of this study are available from the corresponding author upon reasonable request.
